# Quantifying variation in the potential for antibody-mediated apparent competition among nine genotypes of the rodent malaria parasite *Plasmodium chabaudi*^☆^

**DOI:** 10.1016/j.meegid.2013.09.013

**Published:** 2013-12

**Authors:** Karen J. Fairlie-Clarke, Judith E. Allen, Andrew F. Read, Andrea L. Graham

**Affiliations:** a Institutes of Evolution, Immunology and Infection Research, School of Biological Sciences, King’s Buildings, University of Edinburgh, Edinburgh, UK; b Centre for Infectious Disease Dynamics, Department of Biology, Pennsylvania State University, University Park, PA, USA; c Department of Ecology and Evolutionary Biology, Princeton University, Princeton, NJ, USA

**Keywords:** *Plasmodium chabaudi*, Cross-reactivity, Within-host competition, Antibody, Mixed infection, Virulence evolution

## Abstract

Within-host competition among parasite genotypes affects epidemiology as well as the evolution of virulence. In the rodent malaria *Plasmodium chabaudi*, competition among genotypes, as well as clone-specific and clone-transcending immunity are well documented. However, variation among genotypes in the induction of antibodies is not well understood, despite the important role of antibodies in the clearance of malaria infection. Here, we quantify the potential for antibodies induced by one clone to bind another (i.e., to cause antibody-mediated apparent competition) for nine genetically distinct *P. chabaudi* clones. We hypothesised that clones would vary in the strength of antibody induction, and that the propensity for clone-transcending immunity between a pair of clones would increase with increasing genetic relatedness at key antigenic loci. Using serum collected from mice 35 days post-infection, we measured titres of antibody to an unrelated antigen, Keyhole Limpet Haemocyanin (KLH), and two malaria antigens: recombinant Apical Membrane Antigen-1 (AMA-1) and Merozoite Surface Protein-1_19_ (MSP-1_19_). Amino acid sequence homology within each antigenic locus was used as a measure of relatedness. We found significant parasite genetic variation for the strength of antibody induction. We also found that relatedness at MSP-1_19_ but not AMA-1 predicted clone-transcending binding. Our results help explain the outcome of chronic-phase mixed infections and generate testable predictions about the pairwise competitive ability of *P. chabaudi* clones.

## Highlights

• We measure antibody responses induced by 9 genotypes of *Plasmodium chabaudi* in mice.
• *In vitro* antigens include an exoantigen and 2 recombinant malaria antigens.
• Parasite genotypes vary significantly in the magnitude of antibody responses induced.
• Cross-reactivity of anti-MSP1_19_ responses is predicted by amino acid homology.
• Differential antibody induction may predict the outcome of within-host competition.

## 1 Introduction

Intraspecific competition among parasites in mixed-genotype infections is expected to affect the evolution of parasite traits and of virulence (degree of harm done to hosts) (Mideo, 2009). Such within-host competition has been demonstrated in a wide range of parasite taxa (e.g., (Balmer et al., 2009; Bashey et al., 2013; Hall and Little, 2013)) and can affect establishment of infection and transmission from the host (Karvonen et al., 2011), the virulence of infection (Balmer et al., 2009) and parasite population structure (Gold et al., 2009). Natural malaria infections often comprise more than one genotype per species (Babiker et al., 1994; Mobegi et al., 2012; Read and Taylor, 2001; Vardo and Schall, 2007). The rodent malaria parasite *Plasmodium chabaudi* has been used to investigate the ecological mechanisms of within-host competition (Bell et al., 2006; de Roode et al., 2005a,b; De Roode et al., 2003; Taylor et al., 1997). For example, direct competition for red blood cells (RBCs) is paramount during the acute phase of infection where parasite population growth is exponential (De Roode et al., 2003). However, parasite dynamics during mixed infection are not always easily explained by resource (exploitation) competition, particularly during the chronic phase (e.g., (Bell et al., 2006; Mideo et al., 2008)). Instead, immune-mediated apparent competition (where one genotype induces an immune response capable of targeting other genotypes; e.g., (Jarra and Brown, 1985)) or facilitation (if one genotype distracts immunological attention from others) may determine the outcome of within-host competition (Barclay et al., 2008; Raberg et al., 2006). Importantly, the direction of natural selection on parasite virulence depends upon the mechanism of competition (Mideo, 2009).

Malaria poses a particularly interesting system for considering immune-mediated apparent competition and facilitation, because mammalian adaptive immunity is capable of exquisite specificity to malaria antigens (Couper et al., 2005; Quin and Langhorne, 2001), including species- and strain-specific immunity (Jarra and Brown, 1985, 1989; Martinelli et al., 2005; Pattaradilokrat et al., 2007), yet the parasites also induce cross-reactive antibodies through polyclonal expansion of B-cells. This proliferation and differentiation of B-cells regardless of their antigen-specificity (Montes et al., 2007) is attributed to disruption of spleen architecture, innate activation of B-cells, and induction of cytokine storms (Achtman et al., 2003; Castillo-Mendez et al., 2007; Muxel et al., 2011). Indeed, induction of cross-reactive immune responses may be a parasite strategy to promote the chronicity of infection (Recker et al., 2004). Although variation among *P. chabaudi* clones in innate immune response induction has been described (Long et al., 2006, 2008), and immunocompromised mice (lacking all T-cells or CD4+ T-helper cells) have been used to test whether the adaptive immune response influences competition between *P. chabaudi* clones (Barclay et al., 2008; Raberg et al., 2006), the potential for cross-reactive antibodies to mediate competition among a wide range of *P. chabaudi* clones has not been assessed.

In this study, we measured variation among nine clones in the induction of cytophilic antibodies, which exhibit a range of specificities and have great functional importance in the system: they block parasite invasion and development within RBC, bind infected RBC (Cavinato et al., 2001) to facilitate uptake and destruction by phagocytes (Mota et al., 1998), interfere with merozoite dispersal following RBC rupture (Bergmann-Leitner et al., 2009, 2006; Li et al., 2001), and are ultimately required for resolution of *P. chabaudi* infection (von der Weid et al., 1996). To study potential variation in polyclonal stimulation of B-cells by the malaria clones, we measured antibodies binding to the “exoantigen” Keyhole Limpet Haemocyanin, or KLH, a large and antigenically complex molecule (Harris and Markl, 1999) that the animals never experienced *in vivo* and is often used to quantify variation in antigen-independent humoral immune potency (e.g., (Star et al., 2007)). To study the induction of clone-transcending antibody, we measured binding of antibodies to two recombinant malaria antigens, Apical Membrane Antigen-1 (AMA-1) and Merozoite Surface Protein-1_19_ (MSP-1_19_). These antigens are both malaria vaccine candidates (Anders et al., 1998; Burns et al., 2004; Crewther et al., 1996; Dodoo et al., 2008; Hensmann et al., 2004) that are known to be polymorphic in *P. chabaudi* (Cheesman et al., 2009; Crewther et al., 1996; McKean et al., 1993). We expected that these polymorphisms may directly predict the ability of antibodies induced by one clone to bind other clones. Together, our measurements of general immune potency and binding capacity for malaria antigens aid prediction of the mode and strength of immune-mediated competition among clones.

## 2 Materials and methods

### 2.1 Experimental infections

*P. chabaudi* clones were originally isolated from thicket rats (*Thamnomys rutilans*) and cloned by serial dilution and passage (Beale et al., 1978). These clones were stored as cryopreserved blood stabilates and passaged through donor mice prior to experimental infection. The clones we used vary in growth rate and virulence (Long et al., 2008; Mackinnon and Read, 1999) and are listed here in order of ascending maximum parasitaemia (percentage of RBCs infected) achieved in our experiments: AS_11943_, CW_175_, DK_116_, DS_1671_, AT_53_, ER_494_, AJ_4787,_ CR_518_, AQ_218,_ (subscript denotes the point in the lineage from which each clone originates; hereafter clones are identified by their two-letter codes, AS, CW, etc.). All the clones used were *P. chabaudi chabaudi* except for DS and DK which belong to the subspecies *P. chabaudi adami*.

Female C57/BL6 mice (Harlan UK), 16–18 weeks of age, were housed in a 12 h:12 h light–dark cycle, and 41B diet (Harlan UK) and drinking water supplemented with 0.05% para-amino benzoic acid (PABA) were provided *ad libitum* (Jacobs, 1964). For each clone, we established infections by intraperitoneal injection of 1 × 10^5^ *P. chabaudi* parasitised red blood cells (pRBCs). We used 5 experimental mice per clone, except for AS where 6 mice were used. Parasitaemia was monitored daily by ×1000 microscopy of thin tail-blood smears stained with Giemsa, as described previously (Mackinnon and Read, 1999). Mice were exsanguinated, under terminal anaesthesia, in the early chronic phase of infection (day 35 post-infection). This timepoint was at least 3 weeks after the resolution of acute infection (the primary peak in parasitaemia) and is expected to reflect maximum antibody production (Quin and Langhorne, 2001), though measurable responses persist for over 6 months (Achtman et al., 2007). Serum was separated using SeraSieve (Hughes and Hughes Ltd) by centrifugation at 13,000 rpm for 10 min and stored at −80 °C. Protocols for this animal work were approved by the UK Home Office.

### 2.2 Antigens and antibody measurement

Three proteins were used in this study: Keyhole Limpet Haemocyanin, or KLH (SIGMA) and two recombinant malaria antigens; Merozoite Surface Protein-1_19_ (MSP-1_19_) and Apical Membrane Antigen-1 (AMA-1). MSP-1_19_ was originally sequenced from *P. chabaudi chabaudi* clone AS and inserted into *Pichia pastoris* vector pIC9K for expression in *P. pastoris* strain SMD1169 as described previously (Hensmann et al., 2004). Apical Membrane Antigen-1 (AMA-1) was originally sequenced, cloned and expressed from *P. chabaudi adami* clone DK. The AMA-1 nucleotide sequence was inserted into *Escherichia coli* vector PQE9 and expressed in the cell line SG13009 (Crewther et al., 1996).

We used Enzyme Linked Immunosorbent Assays (ELISA) to measure antigen binding of serum immunoglobulin (Ig) isotypes IgG1, IgG2a, IgG2b and IgG3 as described previously (Fairlie-Clarke et al., 2010), with the following adjustments. Serum samples were added in a series of doubling dilutions (1/100 to 1/204800) using TBST as diluent. Antibodies that bound antigen were detected with isotype-specific hrp-conjugated goat anti-mouse antibodies (Southern Biotech: IgG1 1070-05 at 1/6000 dilution, IgG2a 1080-05 at 1/200, IgG2b 1090-05 at 1/4000 and IgG3 1100-05 at 1/1000). Antibody titres were calculated as the reciprocal of the greatest dilution at which the O.D. was greater than the mean plus 2 standard deviations of the O.D values for uninfected control samples binding that antigen at 1/100 dilution. Although all four isotypes were measured results are only presented for the most functionally relevant IgG2a.

### 2.3 Sequencing and bioinformatics

For each clone, when a suitable level of parasitaemia (minimum 5% parasitised RBCs) was observed in the donor mice, we collected 10 μl of whole blood from the tail in an excess of citrate saline (500 μl) and centrifuged at 13000 rpm for 5 min. The resulting pellet was stored at −80 °C. We extracted genomic DNA using the Instagene DNA preparation kit for whole blood (BIO-RAD Cat no. 7326211) according to the manufacturer’s instructions. Polymerase Chain Reaction (PCR) was used to amplify fragments of *msp-1* and *ama-1*, as follows. 8 μl of 1/100 dilution of genomic DNA was used as a template in a 20 μl PCR reaction in combination with 0.1 mM final concentration of the forward and reverse primers. Primers were designed to amplify a 350 base pair fragment of *msp-1* and a 205 base pair fragment of *ama-1*. The region of MSP-1_19_ amplified for the 9 clones was aligned with the AS sequence for *msp-1_19_*, GenBank accession number L22982.1 (nucleotide 4907–5257). For *ama-1*, we used the DK sequence with Genbank accession number U49745 (nucleotide 137–342).

A Taq enzyme High Fidelity PCR Master kit (Roche) was used for amplification of genomic DNA with the programmed temperature profile (95 °C, 1 min; 35 cycles of 94 °C, 45 s; 55 °C, 45 s; 68 °C, 3 min; 72 °C, 5 min). PCR amplification products were detected via 1% agarose gel electrophoresis. Double stranded DNA (dsDNA) was purified directly from the PCR reaction using QIAquick PCR purification Kit (Qiagen) according to the manufacturer’s protocol. A Prism BigDye Terminator and Cycle Sequence Kit Version 3.1 (ABI) were used for the sequencing reaction with 10 ng/μl of dsDNA and 3.2 pmol/μl final concentration of the appropriate primer. The University of Edinburgh School of Biological Sciences Sequencing Service ran the products on an ABI 3730 capillary sequencer. The resulting nucleotide sequences were aligned using MacVector 7.2.3 to determine the percentage amino acid identity, for AMA-1 or MSP-1_19_, amongst the 9 *P. chabaudi* clones. Our order of genetic relatedness for MSP-1_19_ based on amino acid sequence homology is largely in agreement with the genetic relatedness predicted by the maximum likelihood phylogenetic tree, generated from a broader analysis of nucleotide diversity in the *msp-1* gene (Cheesman et al., 2009).

### 2.4 Statistical analysis

The serial dilution of serum to calculate antibody titre produces ordinal data, which were log_10_ transformed to normalize for linear modelling (Grafen and Hails, 2007). All analyses were carried out using statistical package JMP 10 (SAS). General linear statistical models allowed us to test whether differences in clone identity, parasitaemia, and/or amino acid homology to the recombinant antigens best explained the variation in antibody induction. Clone was included as a fixed factor and its ability to predict antibody titre was formally evaluated with maximum parasitaemia (Max%P) as a covariate, plus an interaction term. Models were minimised as described previously (Fairlie-Clarke et al., 2010). When clone was a significant predictor, all pair-wise Tukey post-hoc tests were carried out to identify pairs of clones that generated significantly different antibody titres. Finally, Ordered Heterogeneity (OH) analysis (Gaines and Rice, 1994) allowed a test of the directional hypothesis that clones which were more homologous (in terms of amino acid sequence) to the source clone for a recombinant antigen would exhibit higher antibody titres to that antigen. The ordered heterogeneity test statistic r_s_P_c,_ was calculated according to the equation r_s_P_c_ = r × (1 − P), where r is the Spearman’s rank correlation coefficient describing the relationship between mean antibody titres and percentage amino acid identity of clones. P is the *P*-value from the analysis of titre with amino acid identity as the predictor variable. The *P-*value for the ordered heterogeneity test statistic (r_s_P_c_) was then calculated by reference to the critical values table in Gaines and Rice (1994). For all tests, cut off for statistical significance was taken as *P *< 0.05, and reported *F* statistics and *P-*values are from the minimal model.

## 3 Results and discussion

Our results demonstrate variation amongst *P. chabaudi* clones in induction of cytophilic antibody and generate predictions about the likelihood of immune-mediated competition among pairs. Whilst natural *Plasmodium* infections may comprise many genotypes, it is common for two genotypes per host to be observed (e.g., (Anderson et al., 2000; Auburn et al., 2012; Manske et al., 2012; Vardo and Schall, 2007)); furthermore, investigating the potential for immune-mediated interactions to occur between pairs of clones represents a tractable first step to studying more complex mixed-genotype infections. We deliberately chose to measure antibodies induced during single-clone infections as this permits exploration of the potential for apparent competition without the complications of a mixed-clone setting.

**Fig. 1:**
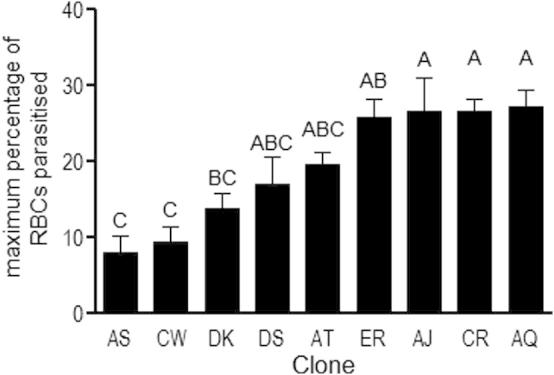
Maximum percentage of red blood cells infected (i.e., parasitaemia) achieved by distinct *P. chabaudi* clones. Figure shows mean and standard error of *n* = 5–6 infected mice per clone. Groups not connected by the same letter denote pairs that are significantly different (*P* < 0.05) according to Tukey’s Pairwise analysis.

**Fig. 2:**
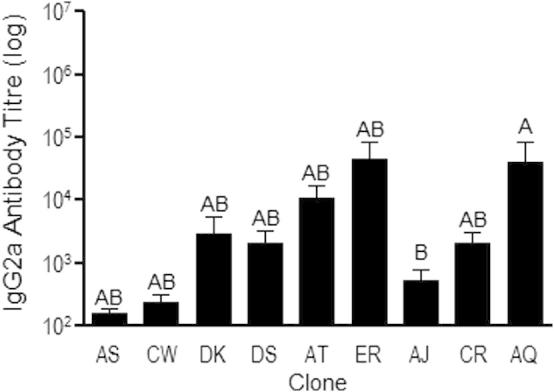
IgG2a antibody response to exoantigen KLH. Antibody titre was calculated as the reciprocal of the greatest dilution at which O.D was greater than the mean plus 2 standard deviations of the O.D for control mouse sera at 1/100 dilution. Figure shows mean and standard error of *n* = 5–6 infected mice per clone. Groups not connected by the same letter denote pairs that are significantly different (*p* < 0.05) according to Tukey’s Pairwise analysis.

Confirming previous reports on the malaria genotypes used in this study (e.g., (Long et al., 2008; Mackinnon and Read, 1999)), clones varied significantly in maximum parasitaemia achieved (Fig. 1; *F*_8,37_ = 8.06; *P *< 0.0001). Clones also varied in polyclonal B-cell induction, as measured by binding to the exoantigen KLH (Fig. 2; *F*_8,28_ = 2.37; *P *= 0.044), and with differential dependence upon parasitaemia (*F*_1,28_ = 0.03; *P *= 0.86 for parasitaemia main effect; *F*_8,28_ = 2.29; *P *= 0.05 for the clone × parasitaemia interaction). This suggests significant variation among clones in their immune potency, or their propensity to non-specifically stimulate a humoral response. The acute phase of malaria infection is characterised by polyclonal B-cell activation (Castillo-Mendez et al., 2007) and therefore the production of antibodies of a wide array of specificities, including highly cross-reactive natural antibodies (Ternynck et al., 1991) and auto antibodies (D’Imperio Lima et al., 1996; Yaffe, 2001), though this is the first evidence of parasite genetic variation in their induction. The role of such natural antibodies in parasite control has not been definitively determined but it is possible that they could interact with conserved parasite antigens (Castillo-Mendez et al., 2007). Greater immune potency of one clone may therefore be associated with an increased likelihood of a clone-transcending response against other clones.

**Fig. 3:**
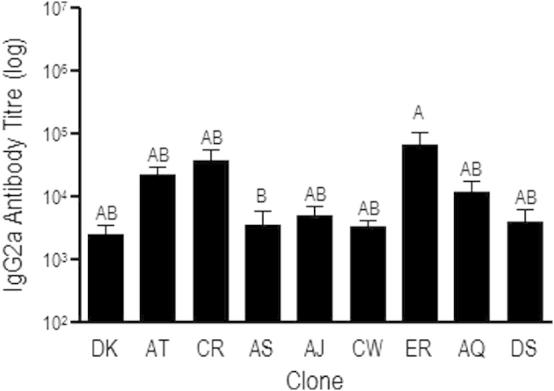
IgG2a antibody response to recombinant AMA-1 antigen from clone DK. Antibody titre was calculated as the reciprocal of the greatest dilution at which O.D was greater than the mean plus 2 standard deviations of the O.D for control mouse sera at 1/100 dilution. Figure shows mean and standard error of *n* = 5–6 infected mice per clone. Groups not connected by the same letter denote pairs that are significantly different (*P* < 0.05) according to Tukey’s Pairwise analysis.

**Fig. 4:**
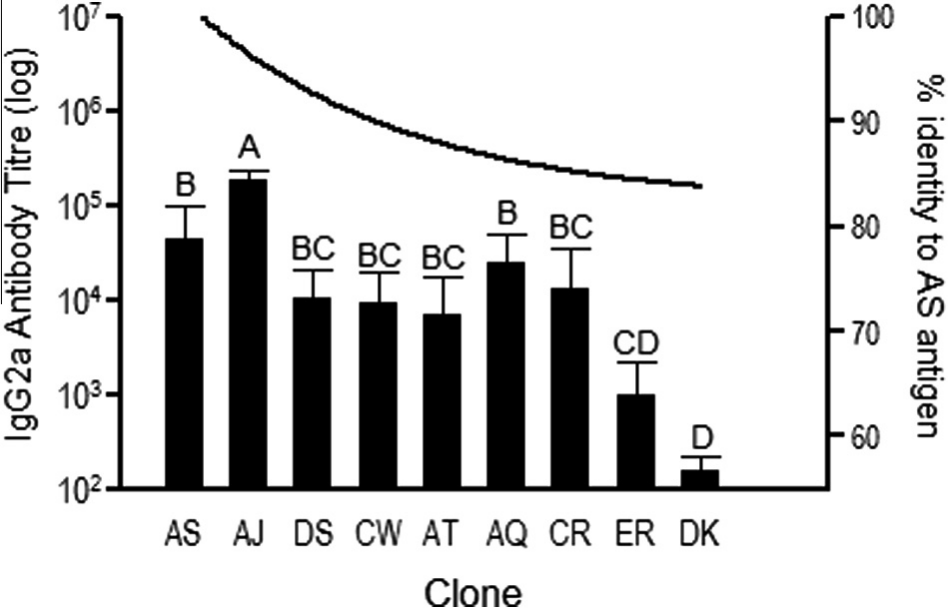
IgG2a antibody response and amino acid homology to recombinant MSP-1_19_ antigen from clone AS. Antibody titre shown as black bars and left *y*-axis was calculated as the reciprocal of the greatest dilution at which O.D was greater than the mean plus 2 standard deviations of the O.D for control mouse sera at 1/100 dilution. Figure shows mean and standard error of *n* = 5–6 infected mice per clone. Groups not connected by the same letter denote pairs that are significantly different (*P* < 0.05) according to Tukey’s Pairwise analysis. The black line and right *y*-axis show the proportion of amino acid sequence homology to the MSP-1_19_ recombinant antigen from clone AS.

We next assessed whether antibody binding to recombinant malaria antigens was explained by malaria genotype, parasitaemia, and/or amino acid sequence homology at antigenic loci. Binding of cytophilic IgG2a antibody to DK-clone’s AMA-1 (DK-AMA-1) varied significantly amongst clones, largely driven by differences between AS and ER but not by differences from DK (Fig. 3, with clones shown in order of decreasing relatedness to the antigenic source clone, DK; *F*_8,37_ = 3.41; *P *= 0.0049). However, neither parasitaemia nor genetic relatedness to DK at the *ama-1* locus were predictive of IgG2a titre (*P* = 0.87 and 0.9, respectively). By contrast, clones induced antibodies capable of binding to MSP-1_19_ in accordance with their amino acid homology to the recombinant antigen. Binding of IgG2a to AS-clone’s MSP-1_19_ (AS-MSP-1_19_) varied significantly amongst clones (*F*_8,37_ = 12.41; *P *< 0.0001), generally in accordance with genetic relatedness at the *msp-1* locus (r_s_P_c_ = 0.67, *P* = 0.01) (Fig. 4, with clones shown in order of decreasing relatedness to the antigenic source clone, AS) but exhibited no relationship with parasitaemia (*P* = 0.26). These findings suggest that when MSP-1_19_ is the target of immune recognition, the ability of host antibodies to bind AS parasites can be predicted by amino acid sequence homology. This was not so for AMA-1. This discrepancy may reflect the greater number of polymorphisms in AMA-1, only some of which are relevant to binding of host antibodies and/or differential immunogenicity of AMA-1 variants (as described in *Plasmodium falciparum*; (Drew et al., 2012; Kusi et al., 2009)). Thus, we predict that clones more similar in amino acid sequence to the MSP-1_19_ source clone AS would generate greater antibody titre to AS-MSP-1_19_, but that amino acid similarity to the AMA-1 source clone DK would not. A genetic method (linkage group selection) for identifying loci that encode target antigens of clone-specific protective immunity likewise highlighted a greater role for *msp-1* (Cheesman et al., 2010; Martinelli et al., 2005) than *ama-1* (Pattaradilokrat et al., 2007).

Our results suggest that MSP-1_19_ amino acid sequence homology (Cheesman et al., 2009) and overall immune potency of competing clones might be used to predict the role of antibodies in competition during mixed-clone infections. For example, we would expect a slow-growing clone to ‘lose’ when in competition with a fast replicating clone with which it shares antigens. Thus, AS should lose to the antigenically similar but faster replicating AJ (and indeed, it does; e.g., (Bell et al., 2006)), but not the antigenically dissimilar CR or ER. Beyond sequence homology, differing immune potency of clones was the best predictor of variation in titre. This suggests that AQ but not AJ could affect AS replication via cross-reactivity.

Our results may also shed light on previously unexplained outcomes of competition among *P. chabaudi* clones, particularly during the chronic phase of infection, when antibody-mediated immune responses may be most influential. For example, our results could reconcile the apparently conflicting results of the studies in immunocompromised mice (lacking T-cells, albeit in different ways) that set out to investigate within-host competition in the absence of immune-mediated apparent competition (Barclay et al., 2008; Raberg et al., 2006). In mixed infection with AS and AJ, competition was alleviated in immunocompromised mice (Raberg et al., 2006) whereas competition was not alleviated between AS and DK (Barclay et al., 2008). We have demonstrated the potential for clone-transcending responses for AS and AJ but not AS and DK. This suggests that competition between AS and DK is unlikely to be immune-mediated so it is perhaps unsurprising that the outcome of mixed infection was not altered in immunocompromised mice.

Of course, the polymorphisms in MSP-1_19_ and AMA-1 are only a fraction of the antigenic variation exhibited by malaria parasites. For example, an extraordinary repertoire of variable surface antigens, encoded for by the *VAR* gene family, are displayed on *P. falciparum* infected RBC. Such antigenic variation and cross-reactivity among variants are expected to promote chronic infections (Recker et al., 2004). A similar gene family (*cir* genes) has been identified in *P. chabaudi* (Lawton et al., 2012), though for the antigens and timescales of our experiments, little role for those variants in driving the variation among clones in immune response induction is expected (Fischer et al., 2003). It would be extremely interesting, though challenging, to determine the potential for immune-mediated competition to be acting through these target antigens. Mosquito transmission of *P. chabaudi* regulates expression of the *cir* genes and also modifies host immune responses (Spence et al., 2013). How immune-mediated apparent competition is affected by vector-transmitted parasites warrants investigation if we are to understand the complexities of natural infections. For example, variation in the number of sporozoites injected by the mosquito and development through the liver stages to the erythrocytic stages may impact the magnitude, specificity and rate of antibody production.

Understanding immune-mediated competition has important implications for vaccine design as well as virulence evolution (Mideo, 2009). IgG2a responses to AMA-1 appear largely similar across clones, suggesting that it has the potential to induce a clone-transcending response. This would be considered desirable for controlling natural infections but may impose strong and uniform immune selection. Recent work by Barclay et al. (2012) has demonstrated that vaccination with AMA-1 leads to the evolution of more virulent DK parasites, which achieve higher parasite densities and are less well controlled by vaccination than the ancestral clone. Whether all *P. chabaudi*, and indeed *P. falciparum*, clones would respond the same way to clone-transcending immune-selection remains to be empirically investigated, as does the potential for immune-mediated competition in vaccinated or non-naïve hosts.

## 4 Disclosure statement

The authors declare no conflict of interest.
